# Quantile normalization of single-cell RNA-seq read counts without unique molecular identifiers

**DOI:** 10.1186/s13059-020-02078-0

**Published:** 2020-07-03

**Authors:** F. William Townes, Rafael A. Irizarry

**Affiliations:** 1 Department of Computer Science, Princeton University, Princeton, NJ USA; 2 Department of Data Sciences, Dana-Farber Cancer Institute, Boston, MA USA; 3 Department of Biostatistics, Harvard University, Cambridge, MA USA

**Keywords:** Gene expression, Single cell, RNA-seq, Normalization, Quasi-UMI

## Abstract

Single-cell RNA-seq (scRNA-seq) profiles gene expression of individual cells. Unique molecular identifiers (UMIs) remove duplicates in read counts resulting from polymerase chain reaction, a major source of noise. For scRNA-seq data lacking UMIs, we propose quasi-UMIs: quantile normalization of read counts to a compound Poisson distribution empirically derived from UMI datasets. When applied to ground-truth datasets having both reads and UMIs, quasi-UMI normalization has higher accuracy than competing methods. Using quasi-UMIs enables methods designed specifically for UMI data to be applied to non-UMI scRNA-seq datasets.

## Background

Single-cell RNA-seq (scRNA-seq) has become a standard tool for measuring gene expression patterns from individual cells. The initial molecule capture and reverse transcription (RT) steps in scRNA-seq protocols result in low quantities of cDNA, so a large number of PCR cycles are needed to produce enough material for measurement. The resulting libraries, that are then sequenced, contain many duplicates of each of the single mRNA molecules extracted from the original cell [1]. To account for this distortion, some protocols include unique molecular identifiers (UMIs), which enable computational removal of PCR duplicates [2]. However, read count datasets generated without UMIs are still commonly used for at least two reasons. First, many public datasets have been produced with non-UMI protocols. Second, current UMI protocols sequence only the 5-prime or 3-prime end of the mRNA molecule and therefore prevent quantification of transcript isoform levels within the same gene [3] or allele-specific expression [4]. An exception to this is the recently proposed Smart-seq3 protocol [5], but few public datasets are yet available from this technique.

In both UMI and read count data, the fraction of zeros per cell is often a dominant source of variation. Not only does the zero fraction strongly correlate with the first principal component, but it also affects the entire gene expression distribution [6]. While the zero fraction could be driven by biological processes such as the cell cycle, this is completely confounded by cell-to-cell differences in capture and RT efficiency, which have nothing to do with underlying biology. For UMI counts, systematic variation introduced by these technical components can be addressed by using multinomial models [7]. However, for read counts, such models are precluded by the additional multiplicative distortions of PCR. Here, we focus on the analysis of read counts from non-UMI protocols such as Smart-seq2 [8]. Note however that read counts (with PCR bias) may also be obtained from UMI protocols if the UMIs are simply ignored when constructing the expression measurements.

The substantial distortions in read counts have motivated the development of sophisticated normalization procedures. One approach is to attempt to transform the data to more closely follow a normal (Gaussian) noise model. For example, log-transformed expression values after normalization by transcripts per million (TPM), scran [9], or SCnorm [10] may be used as input to principal component analysis (PCA) which implicitly assumes Gaussian noise. However, due to the large number of zeros in scRNA-seq, log transformation of normalized counts requires a pseudocount, which introduces substantial bias [11]. The resulting distributions can be far from Gaussian, even for UMI count data [7]. In contrast, the *census counts* method transforms read counts and attempts to match the underlying UMI distribution based on the key observation that the mode of the nonzero UMI count distribution is typically one [1]. Rather than matching a normal distribution, this approach needs only to remove PCR bias to be effective. The resulting census counts can be analyzed as if they were UMI counts by methods specifically developed for UMI data [7, 12]. Census count normalization relies on a complex mechanistic model of scRNA-seq biochemistry and applies a linear transformation [1]. However, due to the nonlinearity of PCR, this approach is inadequate for removing bias.

Here, we present quasi-UMIs (QUMIs), a normalization technique for scRNA-seq read counts that, like census counts, attempts to match the UMI count distribution. Our approach differs from census counts in that we apply quantile normalization rather than a linear transformation, producing a discrete distribution. In general, quantile normalization forces all cells to follow a specific *target distribution*. The most widely implemented version of quantile normalization generates the target distribution by averaging over empirical distributions from the data [13]. In the case of scRNA-seq, however, we know that if we could remove PCR duplicates from read counts, we would obtain UMI counts, which have a markedly different distribution from any of the empirical read count distributions [7]. We therefore use the characteristics of UMI counts as a guide to construct the QUMI target distribution such that it will approximate a true UMI count distribution. Specifically, we fit Poisson-lognormal models to seven public datasets from different tissues, species, and UMI protocols. The Poisson-lognormal distribution has a heavy tail that approximates a power law, and power laws have been observed previously in gene expression [14, 15]. This target QUMI distribution depends on a single shape parameter and makes no assumptions about biochemical mechanisms. On three independent benchmark datasets where both read and UMI counts were available, we transformed read counts to QUMIs and census counts. We assessed accuracy by computing distances between normalized read counts and the true UMI counts. QUMIs had higher accuracy than census counts and read counts. When read counts are affected by gene-length bias [16], TPMs can be used as input to QUMI normalization. Finally, on two datasets without UMIs, using QUMIs combined with a UMI count-based dimension reduction reduced batch effects and increased detection of biological groups.

## Results and discussion

### Datasets

We used thirteen public scRNA-seq datasets (Table 1). For the seven training datasets, we only obtained UMI counts. For the three test datasets and the differential expression dataset, we obtained both UMI counts and read counts. Finally, for the two prediction datasets, we obtained only read counts. We refer to each dataset using the first author’s last name.

**Table 1:** Single-cell RNA-seq datasets used

First author	Year	Species	Tissue	Protocol	Cells	Use
Cao [17]	2017	*C. elegans*	Several	sci-RNA-seq	32,061	Train
Clark [18]	2019	*M. musculus*	Retina	10x chromium V2	7680	Train
Grun [19]	2016	*H. sapiens*	Pancreas	CEL-Seq	1726	Train
Klein [20]	2015	*M. musculus*	Embryonic stem cells	inDrops	2717	Train
Schiebinger [21]	2019	*M. musculus*	Induced stem cells	10x chromium V2	14,925	Train
Zeisel [22]	2015	*M. musculus*	Brain	STRT	3005	Train
Zhang [23]	2019	*H. sapiens*	Synovial/monocytes	CEL-Seq 2	10,001	Train
Macosko [24]	2015	*M. musculus*	Retina	dropseq	7581	Test
Tung [25]	2016	*H. sapiens*	Induced stem cells	SMARTer	564	Test
Zheng [26]	2017	*H. sapiens*	Monocytes	10x gemcode	2612	Test
Vieira Braga [27]	2019	*H. sapiens*	Lung	dropseq	261	DE
Patel [28]	2014	*H. sapiens*	Glioblastoma	Smart-seq2	430	Predict
Segerstolpe [29]	2016	*H. sapiens*	Pancreas	Smart-seq2	1554	Predict

Training data contained only UMI counts. Test and differential expression (DE) data contained UMI counts and read counts. Prediction data contained only read counts

### Current normalization methods inadequate for scRNA-seq read counts

**Fig. 1:**
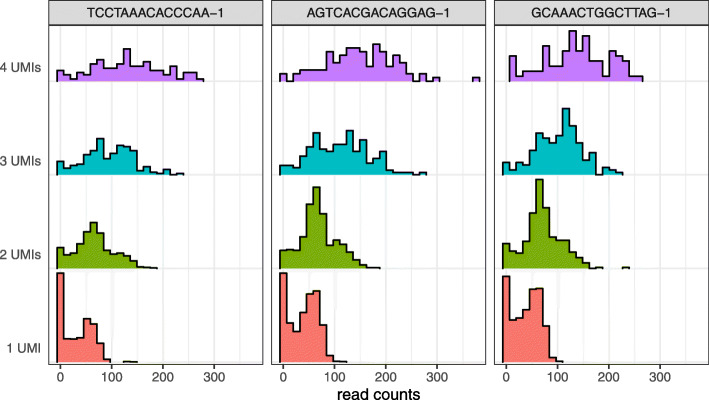
PCR amplification produces a wide range of read count values originating from genes represented by small UMI count values in the Zheng dataset. Each panel is a cell. Color indicates UMI count value

**Fig. 2:**
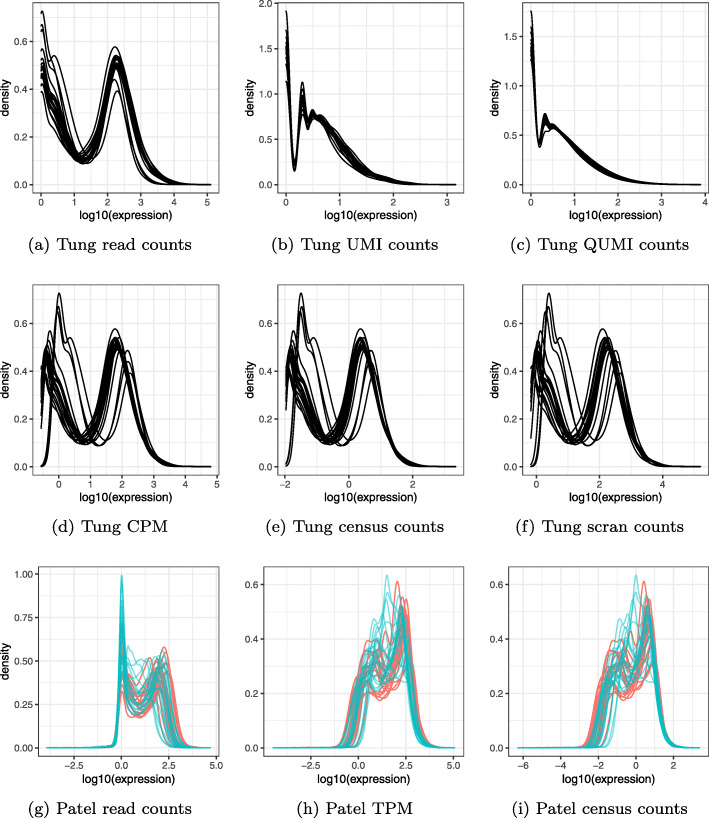
Current normalizations are inconsistent with UMI counts and do not remove distributional variation across biological replicates and batches. **a** Kernel densities of nonzero read count values for 20 random cells from individual NA19098, replicate r3 of the Tung dataset. **b** UMI counts from the same cells as **a**. **c** Quasi-UMI counts (Poisson-lognormal with shape 2.0) computed from read counts of the same cells. **d** As **a** but normalized to counts per million. **e** As **a** but normalized to census counts. **f** As **a** but normalized with scran. **g** Kernel densities of nonzero read count values for 20 random cells from each batch of tumor MGH26 in the Patel dataset. Color indicates batch. **h** As **g** but normalized to transcripts per million. **i** As **g** but normalized to census counts

We explored the effects of normalization on read counts from both UMI and non-UMI protocols. The variability introduced by PCR resulted in hundreds of genes with read counts above 100 that mapped back to less than five UMI counts, in some cases just one (Fig. 1). Current normalization methods such as transcripts or counts per million (TPM, CPM) and census counts apply linear transformations to read counts from non-UMI protocols, which preserve the PCR distortions and result in variable distributions even when the data are generated with the same cell type [25] (Fig. 2a, d–f). Different distributions can be observed when data are processed in different batches (Fig. 2g–i) which can then lead to apparent differences in the low-dimensional representations used, for example, to discover new cell types [6]. For example, the Patel dataset [28] consists of five glioblastoma tumors, with one of these processed in two batches. Current normalizations do not remove the substantial variation in distributions between the batches (Fig. 2h, i). Since not only the scale but also the shape of the distribution of expression values is highly variable between cells of the same biological condition, normalization based on linear transformation is insufficient.

### UMI counts fit by Poisson-lognormal distribution

**Fig. 3:**
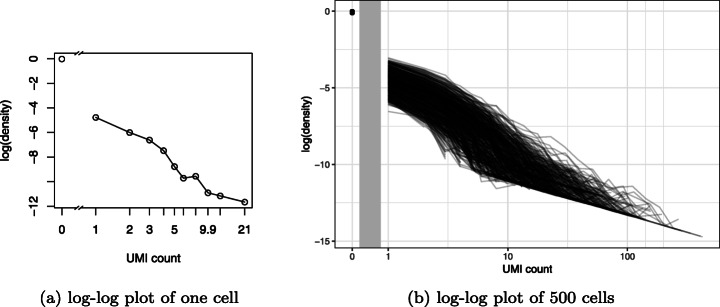
Log-log plots reveal monotonically decreasing, heavy-tailed distributions in UMI counts. **a** Log-log plot of UMI counts from cell cele-002-090.AATCATACGG in the Cao dataset. **b** As **a** but with 500 random cells. Vertical gray bar indicates discontinuity due to horizontal axis log scaling

To quantile normalize read counts to match UMI counts, we identified the qualitative characteristics of the target UMI count distribution. Due to the heavy tail of UMI counts, log-log plots [30] are an effective way to visualize their distribution (Fig. 3a). Log-log plots are essentially histograms with both axes log transformed, and if the right tail of the distribution appears linear, it is suggestive of a power law distribution [31]. Stacking log-log plots for 500 randomly chosen cells, we observed a monotonic decreasing trend for all cells but with substantial variability in the observed proportions for each UMI count value (Fig. 3b). Consistent with [1], the most prevalent nonzero value was one.

A recent survey of a wide variety of datasets found that ostensible power law relationships are better described by lognormal distributions [32]. We therefore considered both the Poisson-Lomax, which has a true power law tail, and the Poisson-lognormal families as candidates for the quantile normalization target distribution. We also compared the negative binomial distribution due to its popularity as a noise model for RNA-seq. Probability mass functions (PMFs) are listed in the “Methods” section.

**Fig. 4:**
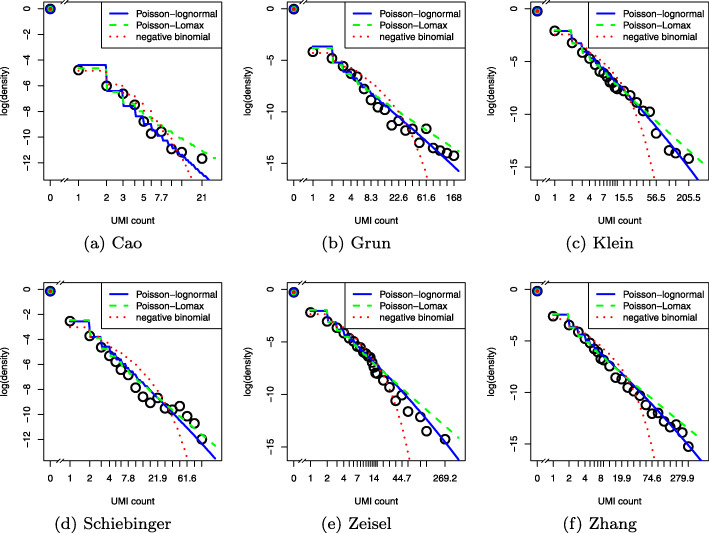
Log-log plots of UMI counts (points) with maximum likelihood fits (curves). **a** Cell cele-002-090.AATCATACGG in the Cao dataset. **b** Cell GSM2142268_ACTGATCG from the Grun dataset. **c** Cell 1146 from the d2 group in the Klein dataset. **d** Cell ACGGCCAGTTATCCGA from sample DiPSC_serum_C2 of the Schiebinger dataset. **e** Cell 1772067059_H11 from the Zeisel dataset. **f** Cell S011_L4Q4_F14 from the Zhang dataset

We first sought to quantify goodness of fit by computing the Bayesian information criteria (BIC) [33] for each cell in the training data, but due to the predominance of zero and low counts, BIC did not clearly distinguish between the three candidate models (Additional file 1: Figure S1). By visualizing randomly chosen cells, we observed the negative binomial was a poor fit to the data, especially for larger counts, due to its lighter tail. Note that this does not contradict the validity of the negative binomial as a noise model, as that corresponds to a conditional probability distribution independent of biological signal, whereas here we are concerned with marginal probabilities that integrate biological signal. While both heavy-tailed distributions fit the training data well overall, the Poisson-Lomax tended to overestimate the probability of high magnitude outliers (Fig. 4). We confirmed this result using a predictive check [34] (Additional file 1: Figure S2); details are provided in the “Methods” section. Furthermore, maximum likelihood estimation of the parameters of the Poisson-Lomax model was numerically less stable. Hence, we focused on the Poisson-lognormal model in our subsequent assessments.

### Quantile normalization of read counts to quasi-UMIs

Assuming the underlying UMI count distribution is Poisson-lognormal, only two parameters are needed to describe each cell: scale and shape. If UMI data are available, these are easily estimated using maximum likelihood (MLEs). However, in read count data without UMIs, this is not possible due to PCR distortion. Conveniently, if the shape parameter is known a priori, the scale parameter can be estimated from the fraction of zeros. This is useful because the zero fraction derived from read counts equals the zero fraction in the UMI counts for the same cell; zero is the only expression value in read count data that is not altered by PCR bias. Therefore, if the shape parameter is assumed known, the target distribution for a given cell can be determined from the read count data. Our method requires the shape parameter to be fixed. To determine reasonable shape parameter values, we computed MLEs for all cells in the training data.

**Fig. 5:**
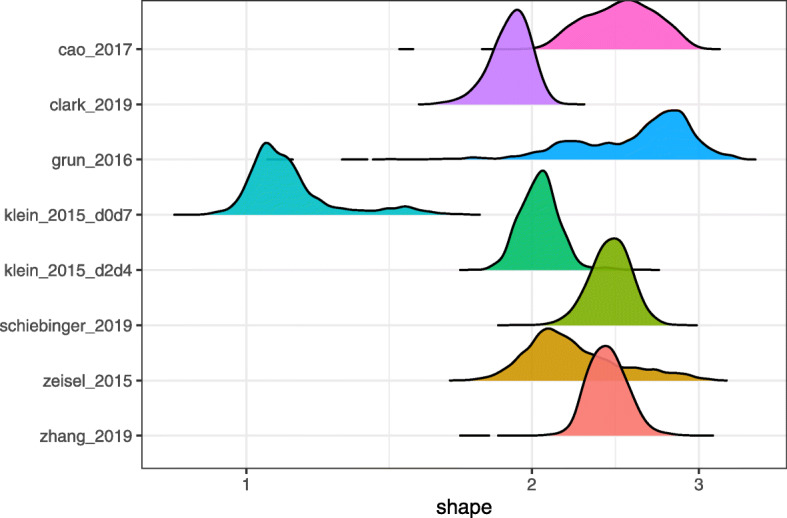
Poisson-lognormal shape parameter maximum likelihood estimates (MLEs) for the training datasets. klein_2015_d0d7 and klein_2015_d2d4 indicate different experimental conditions within the same dataset

The shape parameters varied both within and between training datasets, with values ranging from 1.0 to 3.0 (Fig. 5). We therefore adopted two alternative strategies for quantile normalization. In the default approach, we globally set the shape to 2.0. In the custom approach, for each test dataset, we identified a training dataset with UMI counts from the same tissue type by searching a comprehensive single-cell database [35]. We then used the median of the MLE distribution across cells in the matched training dataset as the shape parameter for all cells in the corresponding test dataset (Additional file 1: Table S1).

While it was necessary to fix the shape parameter before applying quantile normalization to the test data, each cell was allowed to have its own scale parameter. If the scale parameters were also held fixed, the QUMI target distribution would be identical for every cell and would predict a constant zero fraction across cells. But this is discordant with the fact that UMI count data exhibit variation in the zero fraction across cells [7]. Since the varying zero fractions in read counts exactly match the zero fractions in underlying UMI counts, it would be inappropriate to alter these correct expression values by normalizing to a global target distribution. Instead, we estimated each cell’s scale parameter directly from the zero fraction in read counts using the method of moments (MOM). A detailed explanation of the estimation procedure is provided in the “Methods” section. Because this approach matched each cell’s zero pattern, only the nonzero read counts needed to be adjusted by the normalization, which improved computational efficiency.

After estimating the scale parameter for each cell, we obtained empirical quantiles (ranks) from read counts and transformed the ranks to QUMI counts by matching to the target distribution’s theoretical quantiles (see the “Methods” section for detailed algorithm). We did not adjust read counts for gene-length bias because this bias is not substantially present in UMI protocols [16].

In terms of computational speed, quasi-UMI normalization is comparable to census [1]. On the full Segerstolpe dataset, which consisted of 18,978 genes and 2209 cells, census normalization took 23 min (0.6 s per cell), whereas computing QUMI counts with a Poisson-lognormal target distribution and shape 2.0 took 14 min (0.38 s per cell). These numbers reflect serial processing, but QUMI normalization is an independent computation for each cell, so straightforward parallelization can enable scaling to massive datasets. We provide R code for this as part of the companion github repository.

### Quasi-UMIs approximate UMIs more closely than census counts

Using the three test dataset UMI counts as ground truth, we compared the accuracy of Poisson-lognormal quasi-UMI (QUMI) counts with census counts and unnormalized read counts. We quantified the accuracy of a normalization method for a given cell by computing the Euclidean distance between the log of the normalized count vector and the log of the UMI count vector. Zero values were omitted from the computation because all of the normalization methods preserved the sparsity structure of the read counts.

**Fig. 6:**
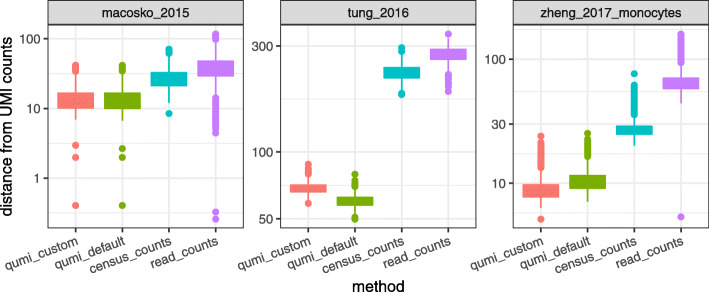
Quasi-UMI counts approximate UMI counts more closely than census counts. QUMI normalization with Poisson-lognormal target distribution was applied to read counts from three datasets. qumi_custom: shape parameter (1.9 for Macosko, 2.4 for Tung and Zheng) set by maximum likelihood fit to matched training data from same tissue type. qumi_default: shape parameter set to 2.0 for all datasets

Across datasets, QUMI counts had the highest accuracy (smallest median distance from UMIs counts), while census counts were more accurate than read counts (Fig. 6). The improvement from using QUMI normalization was most dramatic on the deeply sequenced Tung dataset. The Macosko and Zheng datasets came from droplet protocols with shallow sequencing, while the Tung data came from a plate protocol. The latter is more similar to non-UMI protocols such as Smart-seq2, suggesting QUMI normalization is likely to be effective in those settings. A visualization of the QUMI count distribution shows its strong similarity to the UMI count distribution (Fig. 2b, c).

The accuracy of QUMI counts was not strongly affected by the choice between default and custom shape parameters. This could be due to the custom parameters being close to the default value for these particular test datasets (Additional file 1: Table S1). As a sensitivity analysis, we repeated the QUMI normalization for all datasets with fixed shape parameter values at the extremes of the training data MLE distributions. While this dramatic misspecification of the shape parameter degraded the accuracy of QUMI counts, the difference was small compared to the difference between QUMIs with any parameter value and census counts (Additional file 1: Figure S3).

In addition to an overall comparison averaging across genes, we examined the effects of competing normalization schemes on gene-level statistics that average across cells. For each normalization method and gene, we computed the average expression and coefficient of variation on the Tung and Zheng test datasets. We compared these to the same statistics computed using UMI counts as a ground truth using M-A plots. The quasi-UMI counts were much more consistent with UMI counts than read counts, scran, or census counts (Additional file 1: Figures S4,S5).

To examine the effect of QUMI normalization on differential expression (DE) analysis, we obtained read counts and UMI counts from the Vieira Braga dataset (Table 1), a dropseq experiment on the human lung [27]. We identified differentially expressed genes between endothelial and ciliated cells from UMI counts as a ground truth, then performed the same DE test on each of the competing normalizations as well as the raw read counts. The quasi-UMI normalization produced *p* values and gene sets most concordant with the ground truth (Additional file 1: Figure S6).

### Quasi-UMIs enable dimension reduction of read counts

Quasi-UMI counts may be analyzed as if they were UMI counts. To illustrate this, we applied quasi-UMI normalization, scran [9], and census [1] to TPM values from the Patel dataset [28]. We used TPM values instead of raw read counts as input because full-length scRNA-seq protocols exhibit gene-length bias [16]. This dataset lacked UMIs and profiled 430 cells from five glioblastoma tumors. One tumor (MGH26) was processed in two batches on two different sequencing machines. These two batches differed in the fraction of zeros [6].

**Fig. 7:**
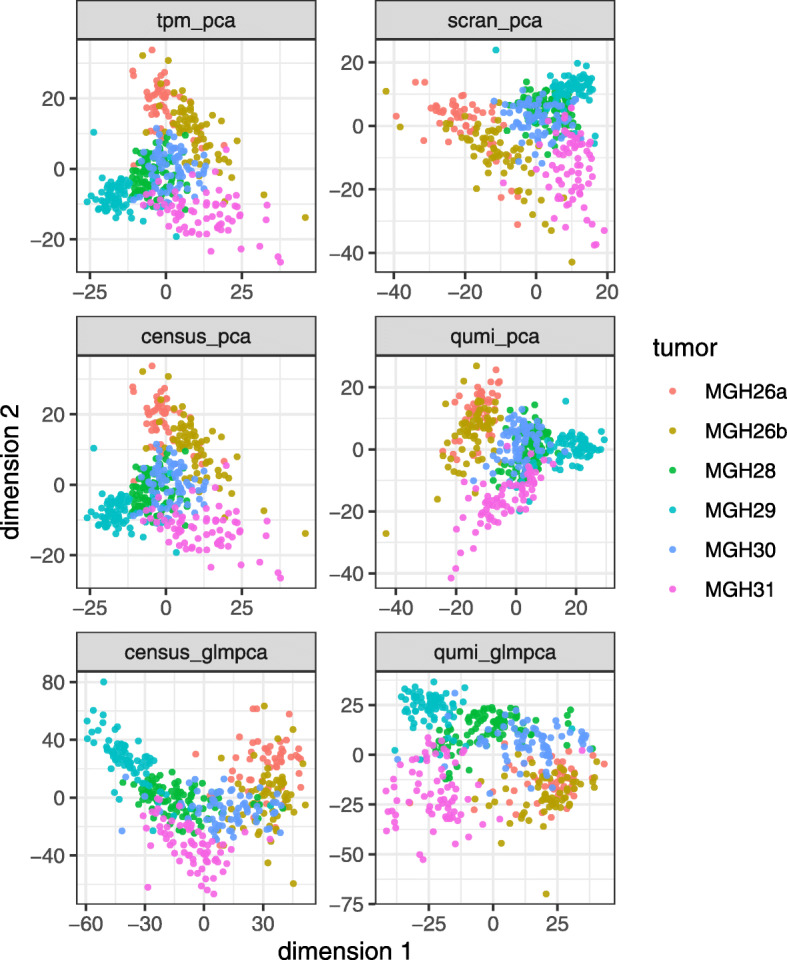
GLM-PCA dimension reduction applied to quasi-UMI counts reduces batch effects in the non-UMI Patel glioblastoma dataset. GLM-PCA was designed for UMI counts. In the top four panels, PCA was applied to normalized counts, and all normalizations failed to merge the two batches of tumor MGH26. In the bottom two panels, GLM-PCA was applied directly to census counts and QUMI counts (Poisson-lognormal with shape 2.0)

We examined the effects of normalization on downstream dimension reduction using principal component analysis (PCA) [36], GLM-PCA [7], and UMAP [37]. Preprocessing is described in the “Methods” section. PCA applied to normalized counts failed to merge the two batches of MGH26 for all normalizations. This was not surprising for QUMI counts since they, like UMI counts, follow a discrete distribution that violates implicit PCA assumptions [7]. In contrast, GLM-PCA, a dimension reduction method specifically designed for UMI counts, when applied to QUMI counts merged the MGH26 batches (Fig. 7). GLM-PCA applied to census counts did not remove the batch effect however. The results were similar when the nonlinear UMAP algorithm [37] was used instead of PCA (Additional file 1: Figure S7). This showed that QUMI counts remove a prominent source of nuisance variation when combined with an appropriate dimension reduction method such as GLM-PCA.

### Quasi-UMIs improve biological resolution of read counts

**Fig. 8:**
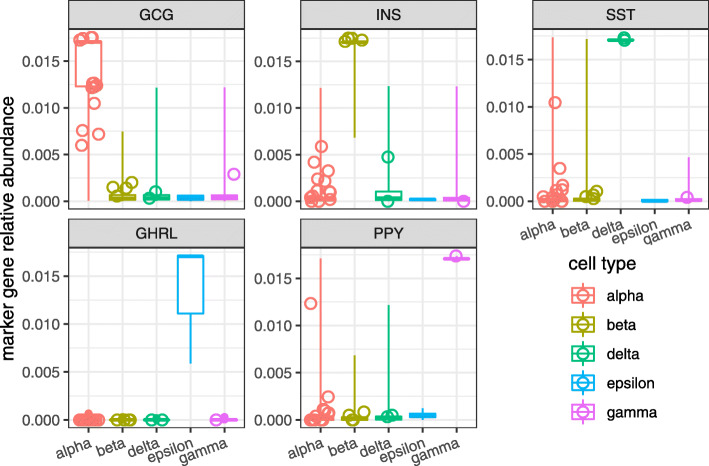
Quasi-UMI normalization improves resolution of pancreatic endocrine cells in the non-UMI Segerstolpe dataset. Each panel represents a marker gene for a specific cell type. Box plots show gene expression levels for cells that were annotated to each cell type by the original authors. Open circles indicate gene expression levels for cells that were unable to be annotated by the original authors. These cells were unambiguously assigned based on a random forest classifier applied to a GLM-PCA dimension reduction of QUMI count values. The overlap between annotated and reclassified cells validates the accuracy of the classification

We examined the ability of QUMI counts to profile a heterogeneous tissue using the Segerstolpe pancreas dataset [29]. The original authors provided annotations for all but 41 of the 1554 endocrine cells. These unclassified endocrine cells were observed as a separate cluster without any clear biological characterization in the original analysis. Using QUMI counts for all genes, we reduced the dimensionality with GLM-PCA to 20 latent factors. We visualized the cells by applying t-SNE [38] to the GLM-PCA factors and observed many of the unclassified cells associated with known clusters (Additional file 1: Figure S8). Building on this exploratory result, we predicted the types of the unknown cells using a random forest classifier fit to the QUMI-derived GLM-PCA features, achieving unambiguous results in 20 out of the 41 cells. We then validated these predictions by comparing the relative abundances of the marker genes in the newly classified cells against the cells that were annotated by the original authors and found high concordance (Fig. 8). This showed that QUMI counts can be used to enhance biological insights in a complex tissue.

## Conclusion

We have shown that UMI counts can be approximated by quantile normalization of read counts to quasi-UMIs (QUMIs) in scRNA-seq. The Poisson-lognormal model fits UMI count data well and can be used as a target distribution for QUMI normalization. However, the conceptual framework is generalizable to any discrete distribution that can be calibrated against UMI data, such as the Poisson-Lomax or two-component mixture models of active and inactive genes [39]. Using test datasets with read counts and UMI counts from the same cells, we confirmed QUMI counts approximate UMI counts more closely than census counts and unnormalized read counts.

QUMI normalization mitigates the distortion of PCR amplification in scRNA-seq protocols that lack UMIs while preserving sparsity. However, just like the use of proper UMIs, it does not normalize differences between cells resulting from variation in efficiency of capture or reverse transcription. This contributes to differences in the zero fraction across cells, which are intentionally preserved in QUMI normalization. These sources of technical variation should be addressed through UMI-specific count models such as GLM-PCA or its approximations using residuals [7, 12].

QUMI counts do not directly account for PCR bias arising from differences in gene length or GC content. These biases are not specific to single-cell protocols and have been addressed in the bulk RNA-seq literature [40]. Since QUMI normalization only requires the rank ordering of genes in each cell along with the fraction of zeros, bias-adjusted TPM values from pseudoaligners [41, 42] can be used as input instead of raw read counts. We followed this approach in analyzing the Patel and Segerstolpe datasets.

A major advantage of QUMI counts is that they can be analyzed as if they were UMI counts. This avoids the need to develop customized methods of dimension reduction and feature selection for the read count distribution. Here, we have focused specifically on scRNA-seq read counts, but traditional bulk RNA-seq read count data is also affected by distortion from PCR amplification. While it may be possible to extend the QUMI framework to bulk RNA-seq data, an appropriate target distribution would need to be identified. This is challenging because a bulk RNA-seq sample, unlike scRNA-seq, is typically a mixture of cell types with unknown proportions. Such a mixture is unlikely to be easily characterized by a simple two-parameter distribution. PCR distortion is also present in read counts from metagenomics experiments [43, 44].

Finally, we caution that QUMIs are not substitutes for proper UMIs. If the latter can be used in an experiment, they will certainly be more effective than QUMIs in removing PCR distortions. QUMI normalization relies on assumptions, such as a fixed shape parameter, which may not be met in certain datasets. Indeed, we observed in the Klein dataset that the shape parameter was not constant across experimental conditions. However, based on our sensitivity analysis, the accuracy of QUMI counts was robust to misspecification of the shape parameter.

## Methods

### Data acquisition and preprocessing

#### Training data (UMI counts only)

The Cao dataset [17] was obtained by following instructions on the authors’ website http://atlas.gs.washington.edu/worm-rna/docs/. The Clark dataset [18] was obtained by following instructions on a companion github repository https://github.com/gofflab/developing_mouse_retina_scRNASeq. The Grun dataset [19] was downloaded from the conquer repository [45] http://imlspenticton.uzh.ch:3838/conquer/. A preprocessed version of the Klein dataset [20] was downloaded from https://hemberg-lab.github.io/scRNA.seq.datasets/mouse/esc/. The Schiebinger dataset [21] was downloaded from GEO accession GSE115943, and only completely differentiated iPSCs were included in the analysis. The Zeisel dataset [22] was downloaded from the authors’ website http://linnarssonlab.org/cortex/, and low-quality cells were removed according to the same criteria used in the original publication. The Zhang dataset [23] was downloaded from ImmPort accession SDY998.

#### Test and differential expression data (UMI and read counts)

The Macosko dataset [24] was obtained by pseudoaligning raw FASTQ files from Sequence Read Archive using Kallisto version 0.45.1 [41] to produce BUS files [46]. We only included sample r6 from this dataset. The Tung dataset [25] was obtained by following instructions on the authors’ website https://jdblischak.github.io/singleCellSeq/analysis/compare-reads-v-molecules.html. The Zheng dataset [26] was obtained by processing the per-molecule information file from https://support.10xgenomics.com/single-cell-gene-expression/datasets/1.1.0/cd14_monocytes. The Vieira Braga dataset [27] was obtained by the same procedure as for the Macosko dataset, except we used Kallisto version 0.46.2.

#### Prediction data (TPMs from read counts only)

The Patel dataset [28] was downloaded from https://github.com/willtownes/patel2014gliohuman. The Segerstolpe dataset [29] was obtained from the scRNAseq Bioconductor R package version 2.0.2.

Scran normalization was applied using version 1.14.6 of the Bioconductor R package. Census counts were obtained using version 2.14.0 of the monocle Bioconductor R package.

### Compound Poisson distributions

The probability mass function (PMF) of a compound Poisson distribution is obtained by placing a prior on the rate parameter of an ordinary Poisson distribution:

$$P(X=x) = \int_{0}^{\infty} \frac{\lambda^{x} e^{-\lambda}}{x!} f(\lambda)d\lambda $$

For the Poisson-lognormal distribution with shape (logarithmic standard deviation) *σ* and scale (logarithmic mean) *μ*, the prior is a lognormal distribution with the same parameters:

$$f(\lambda) = \frac{1}{\lambda\sigma\sqrt{2\pi}}\exp\left\{-\frac{(\log \lambda - \mu)^{2}}{2\sigma^{2}}\right\} $$

For the Poisson-Lomax distribution with shape (power law tail index) *α* and scale *θ*, the prior is a Lomax (shifted Pareto) distribution with the same parameters:

$$f(\lambda) = \frac{\alpha}{\theta}\left(1+\frac{\lambda}{\theta}\right)^{-(\alpha+1)} $$

Let *m* and *v* represent the mean and variance of a given prior distribution in a compound Poisson model. The marginal mean of the compound Poisson is also *m*, and the marginal variance is *m*+*v*. For example, for the Poisson-lognormal, the mean is exp(*μ*+*σ*^2^/2) and the variance is *m*+(exp(*σ*^2^)−1)*m*^2^. The Poisson-Lomax distribution has such a heavy tail that its moments are only finite in certain parameter regions. If *α*>1, then the mean is *θ*/(*α*−1). If *α*>2, then the variance is $m+\frac {\alpha }{\alpha -1}m^{2}$. The quadratic variance function in both families is shared with the negative binomial distribution, so none of them can be distinguished based on coefficient of variation. The Poisson-lognormal has a strictly heavier tail than negative binomial, and Poisson-Lomax has a strictly heavier tail than Poisson-lognormal.

For Poisson-lognormal, we evaluated the PMF using the R package sads. For Poisson-Lomax, we evaluated the PMF by using 1000 numerical quadrature points. For each cell in the training datasets, we obtained maximum likelihood estimates (MLEs) of compound Poisson model parameters (shape, scale) by numerical optimization using the R function optim. The median of the shape parameter distribution across cells was then used to calibrate the quasi-UMI target distribution in the test and prediction datasets.

### Goodness of fit to training data by predictive checks

For each cell, we simulated a vector of gene expression using the fitted MLE parameters. We then identified the maximum expression value (count) for each simulated cell. The test statistic was defined as the log of the ratio of the simulated maximum divided by the observed maximum in the original UMI counts. Each cell then had its own test statistic. If the statistic was close to zero, that indicated the fitted model was well calibrated to the tail of the UMI count data. We therefore computed histograms of the test statistic’s distribution across all cells in each training dataset and compared how close the distribution was to a target of zero.

### Computing quasi-UMIs from read counts

#### Method of moments estimates from zero fractions

For each cell in the test data, we obtained a target quasi-UMI distribution by estimating the cell-specific scale parameter from the empirical zero fraction in read counts using the method of moments (MOM). Specifically, let *f*(*x*;*μ*_*i*_) be the Poisson-lognormal probability mass function (PMF) with fixed shape parameter *σ* and unknown cell-specific scale parameter *μ*_*i*_. For a given cell *i*, the theoretical probability of a zero is *f*(0;*μ*_*i*_) (a function of *μ*_*i*_ only since *σ* is fixed). The empirical probability of zero is simply the fraction of genes with zero read counts in that cell, which we denote with $\hat {p}_{0i}$. A MOM estimate of *μ*_*i*_ is obtained by finding a root of the function $f(0;\mu _{i})-\hat {p}_{0i}$ with respect to *μ*_*i*_.

#### Quantile normalization

Once a target distribution for an individual cell was determined, we computed the log of the theoretical CDF by cumulatively applying the log-sum-exp transformation to the log-PMF function, which provided numerical stability. We then renormalized the probability distribution to exclude the zero value since zero values in read counts result from UMI counts of zero and do not need to be adjusted. This resulted in a table with positive integer indices providing the quasi-UMI count value and corresponding zero-truncated CDF values indicating the probability of a random variable with the target distribution falling below that value, conditional on it being nonzero. We then converted the vector of read counts (or TPMs for Smart-seq2 data) from all genes in the cell to empirical quantiles (ranks). Each gene was then aligned to a CDF bin based on its rank. For example, if the zero-truncated CDF had values of 0.8 at 1 and 0.9 at 2, the first 80% of genes with lowest nonzero read count values would be assigned QUMI value of 1 and the next lowest 10% of genes would be assigned QUMI value of 2. Typically, a single gene was placed into the highest QUMI bin due to the heavy tail of the target distribution.

### Differential expression

For the Vieira Braga dropseq dataset (Table 1), we normalized the read counts to QUMI counts based on the Poisson-lognormal distribution with shape parameters of 1 and 2, as well as with the census method [1]. We selected 159 endothelial and 102 ciliated cells from donor 3 (a male nonsmoker). We retained 12,761 genes that were nonzero in at least one cell out of the 261 total cells. Note, this filtering did not discard genes that were entirely zero in one of the two cell types. We computed *p* values using Fisher’s exact test for each gene for each normalized count matrix as well as the unnormalized read counts. We used the *p* values computed from the UMI counts as a ground truth and computed three distance metrics for each of the other normalizations using the R package amap. First, we computed the Manhattan distance between the vector of *p* values. Next, we computed the Kendall distance which is based on ranks rather than numeric values of the *p* values. Finally, we used the Holm method [47] to adjust the *p* values for multiple comparisons and identified sets of differentially expressed genes at significance <0.05 for each normalization method. We then computed the Jaccard distance between these sets and the set identified using UMI counts.

### Dimension reduction and classification of read count datasets

Since neither QUMI nor census normalization of TPM values removes cell-to-cell variation in total counts, we divided the normalized counts by the total counts of each cell, then multiplied all values by the median of the total count distribution across cells. This ensured all cells had the same total counts. We then centered each feature (gene) to have zero mean and scaled to have unit standard deviation prior to running PCA or nonlinear dimension reductions such as tSNE [38] or UMAP [37]. Negative binomial GLM-PCA, which automatically adjusts for differences in total counts by using an offset term, was always applied directly to untransformed census or QUMI counts.

For the Patel dataset, scran, census, and QUMI normalizations were applied to TPM values. The QUMI target distribution was set to Poisson-lognormal with shape 2.0. Only the 5685 genes used by the original authors were included as input to both dimension reduction algorithms. We directly visualized the cells in two dimensions using PCA, GLM-PCA, and UMAP.

For the Segerstolpe dataset, after excluding non-endocrine cells, QUMI normalization (Poisson-lognormal with shape 2.0) was applied to TPM values and GLM-PCA was run on all 18,301 genes that had at least one nonzero count value across all cells. The number of dimensions was set to 20, and categorical batch indicator variables were regressed off from the latent factors using a linear model. We visualized the endocrine cells using t-SNE with the 20 batch-corrected GLM-PCA factors as input (normally t-SNE uses PCA with 50 dimensions). We trained a random forest classifier on the 20 GLM-PCA features using labels provided by the original authors indicating cell types. We then used the classifier to predict the labels for the 41 cells the original authors were not able to cluster. We defined an unambiguous classification as one where the predicted probability of the assigned class was >0.5. For each cell type, the original authors validated the cluster identity using a marker gene. We therefore validated our classification by comparing the QUMI count relative abundances of each marker gene for newly classified cells versus the cells that were annotated by the original authors in the same category.

## Supplementary information

**Additional file 1** Contains supplementary figures S1–S8, and table S1.

**Additional file 2** Review history.
