# Supplementary material for: Quantile normalization of single-cell RNA-seq read counts without unique molecular identifiers
Source: Genome Biol. 2020 Jul 3;21:160. doi: 10.1186/s13059-020-02078-0 (PMC7333325; doi:10.1186/s13059-020-02078-0)
Supplement: Supplementary file 1 — Additional file 1 Contains supplementary figures S1–S8, and table S1. [file 13059_2020_2078_MOESM1_ESM.pdf]

Supplemental Figures and Tables

Table S1: Custom Poisson-lognormal shape parameters for each test dataset were obtained by using matched training data from the same tissue type.

training data	test data	tissue	Poisson-lognormal shape
Clark_2019	Macosko_2015	retina	1.9
Schiebinger_2019	Tung_2016	induced stem cells	2.4
Zhang_2019	Zheng_2017	monocytes	2.4

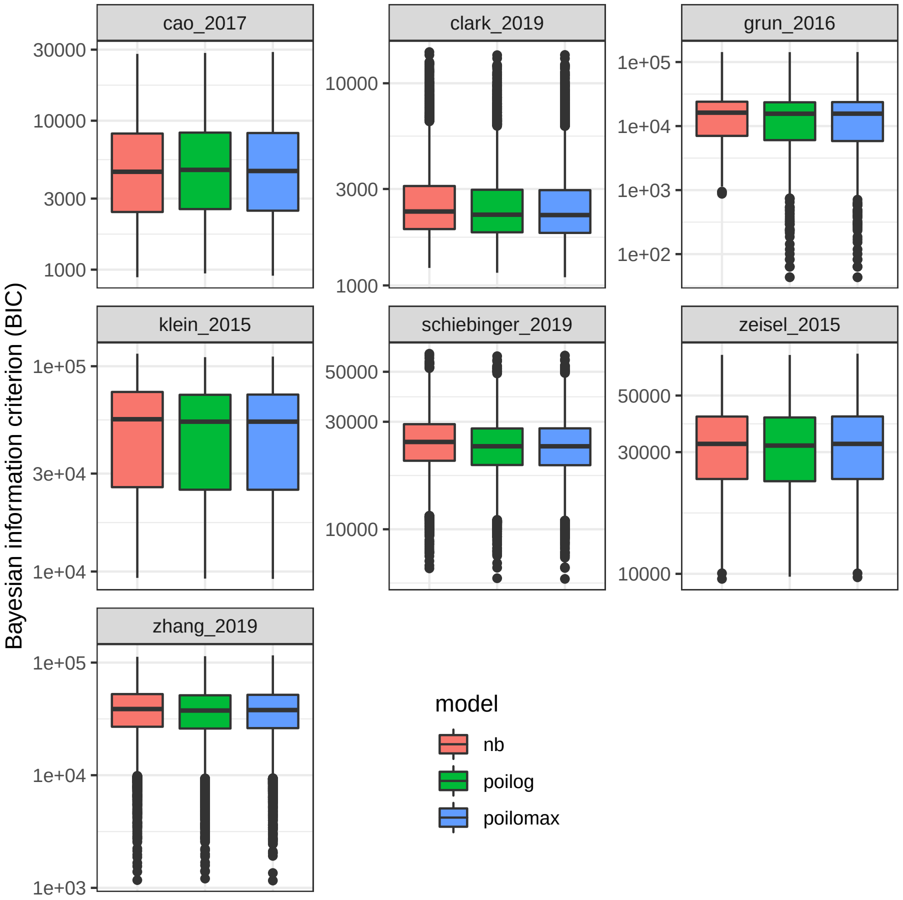

Figure S1: Global statistics such as Bayesian information criterion (BIC) do not clearly distinguish goodness of fit of maximum likelihood estimates from three discrete distributions when applied to training data UMI counts. nb: negative binomial, poilog: Poisson-lognormal, poilomax: Poisson-Lomax.

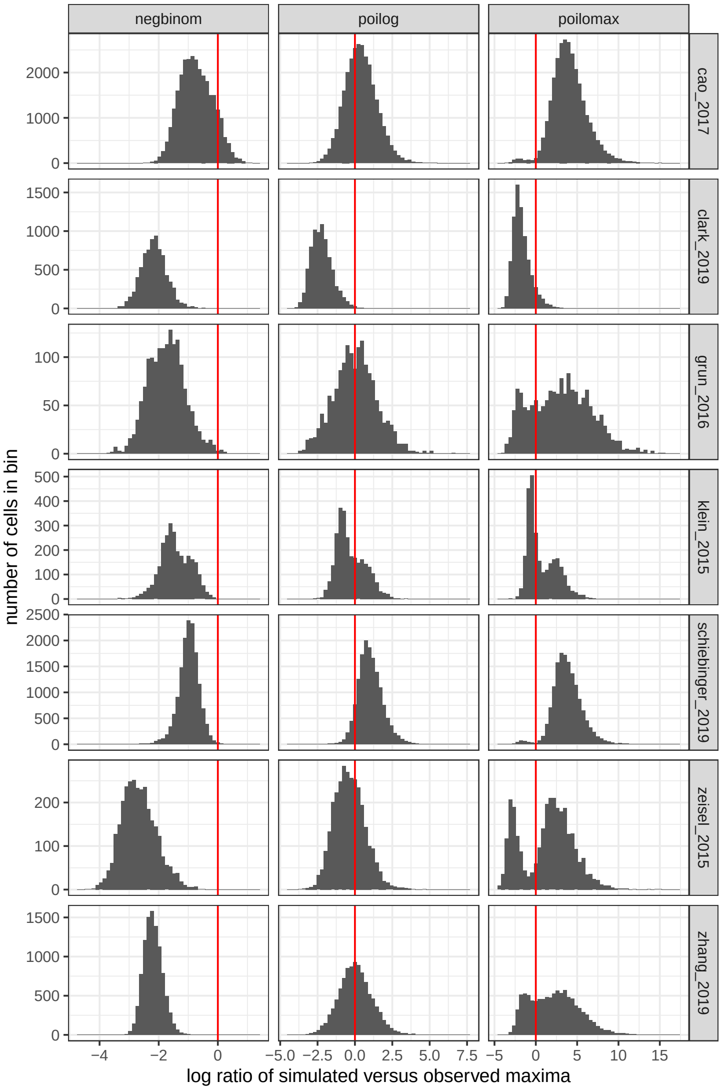

Figure S2: Poisson-lognormal model best fits training data when measured by a predictive check. Goodness-of-fit was quantified by the discrepancy between the maximum of a simulated count vector for each cell and the maximum of the UMI counts from the same cell. Using the maximum as a predictive check emphasizes goodness-of-fit in the tail of the distribution. Vertical red line at zero indicates an optimal fit (exact prediction of the true maximum). The negative binomial model (left column) underpredicts the maximum due to a light tail. negbinom: negative binomial, poilog: Poisson-lognormal, poilomax: Poisson-Lomax.

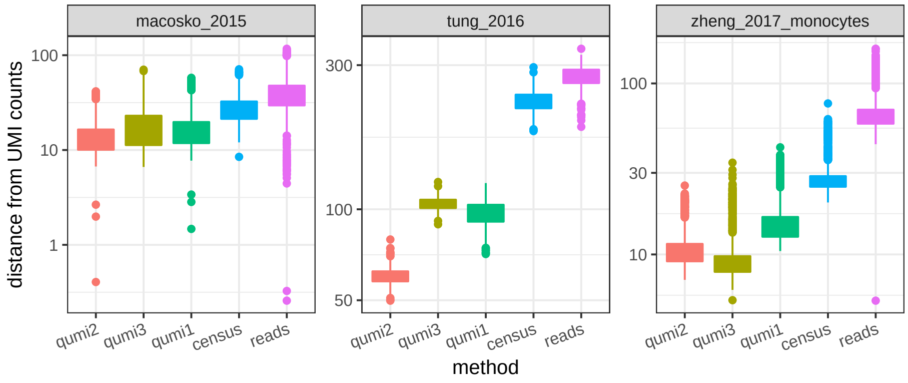

Figure S3: Quasi-UMI counts are robust to variation in the shape parameter. Quantile normalization with Poisson-lognormal target distributions was applied to read counts from three datasets. qumi1, qumi2, qumi3: QUMI counts with shape parameters 1.0, 2.0, 3.0. census: census counts. reads: read counts.

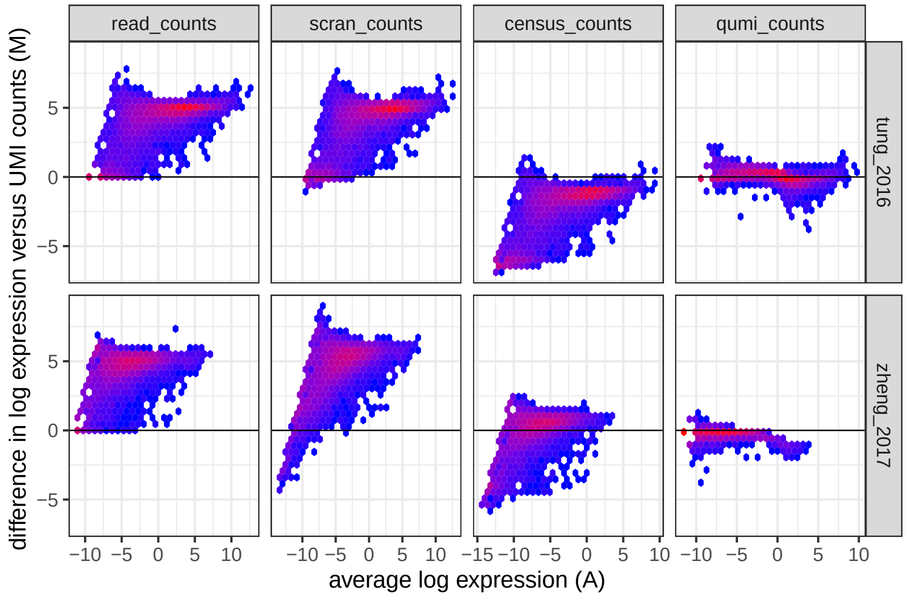

Figure S4: Quasi-UMI counts are most consistent with UMI counts in terms of average expression of individual genes across cells. Each column is a different normalization. Each row is a different dataset. Number of genes: 18,378 for Tung and 14,175 for Zheng. Blue indicate a low number of genes in a tile, red indicates a high number of genes.

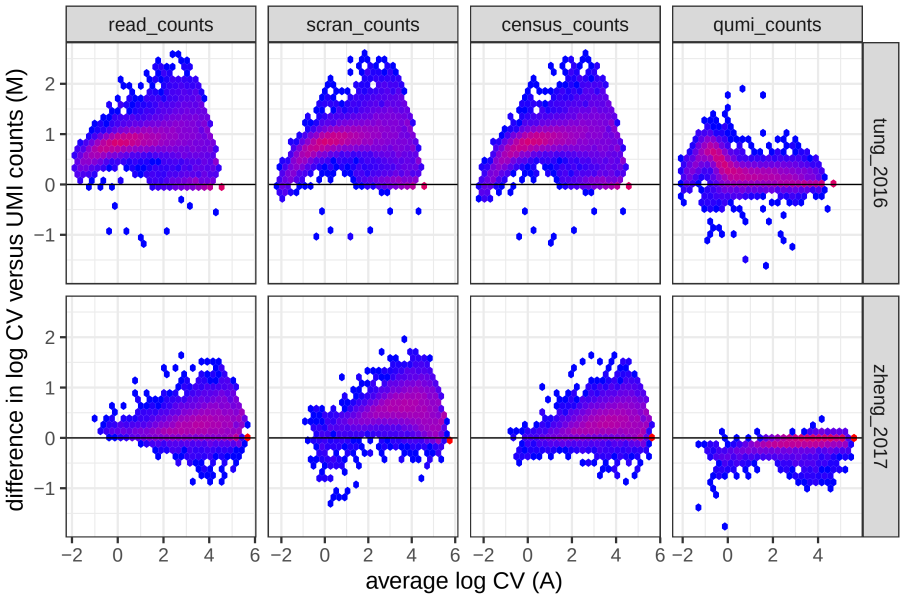

Figure S5: Quasi-UMI counts are most consistent with UMI counts in terms of coefficient of variation of individual genes across cells. Each column is a different normalization. Each row is a different dataset. Number of genes: 18, 378 for Tung and 14, 175 for Zheng. Blue indicate a low number of genes in a tile, red indicates a high number of genes.

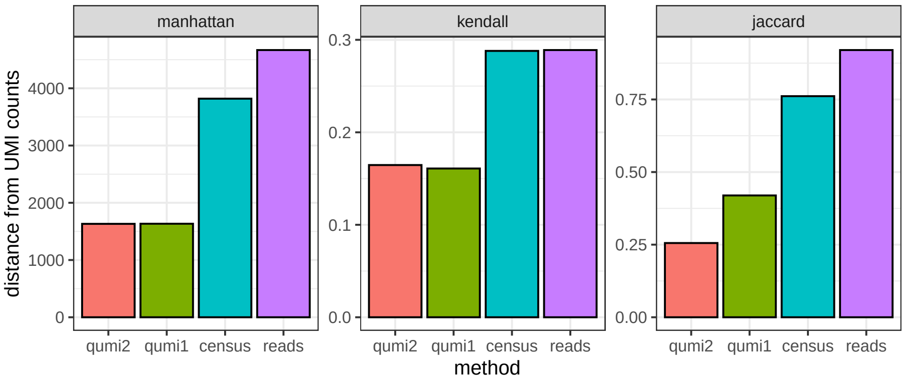

Figure S6: Differential expression (DE) on quasi-UMI counts best approximates results from true UMI counts. Fisher's exact test was applied to ciliated and endothelial cells from a single drop-seq sample of human lung in the Vieira Braga dataset. Manhattan (left) and Kendall rank (center) distance metrics were computed between p-values from UMI counts and each competing normalization. The Jaccard distance (right) quantified the discrepancy between gene sets identified as significantly DE at significance .05 after multiple testing adjustment. Smaller distances indicate closer agreement with results from UMI counts. qumi1,qumi2: Poisson-lognormal QUMI counts with shape parameters of 1.0 and 2.0 respectively, census: census counts, reads: read counts.

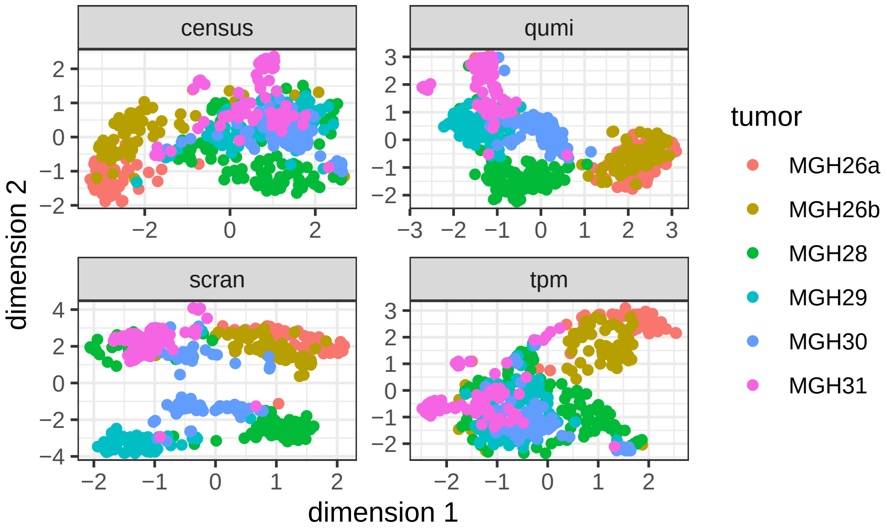

Figure S7: UMAP dimension reduction applied to normalized read counts from the Patel glioblastoma dataset. Each panel is a different normalization. QUMI counts were computed using the Poisson-lognormal distribution with shape 2.0.

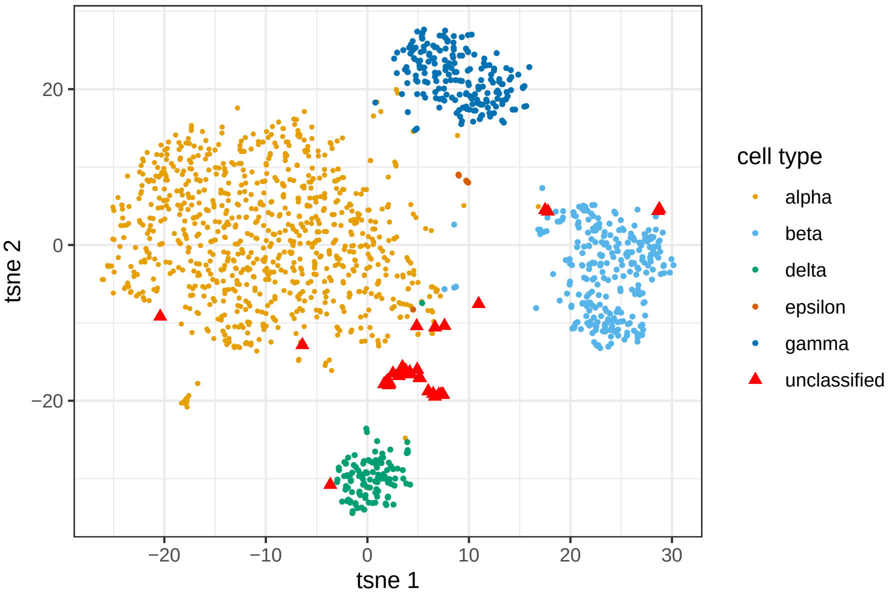

Figure S8: Quasi-UMI normalization improves tSNE resolution of pancreatic endocrine cells in the non-UMI Segerstolpe dataset. Colored points indicate cells that were annotated by original authors. Red triangles indicate cells that were unable to be categorized by original authors. Quasi-UMI counts of all genes were compressed to 20 latent factors using GLM-PCA. These factors were then visualized using tSNE. The proximity of many previously unannotated cells to known clusters suggested they could be reclassified.
