# Supplementary material for: Quantile normalization of single-cell RNA-seq read counts without unique molecular identifiers
Source: Genome Biol. 2020 Jul 3;21:160. doi: 10.1186/s13059-020-02078-0 (PMC7333325; doi:10.1186/s13059-020-02078-0)
Supplement: Supplementary file 2 — Additional file 2 Review history. [file 13059_2020_2078_MOESM2_ESM.docx]

Review History

**First round of review**

**Reviewer 1**

**Were you able to assess all statistics in the manuscript, including the appropriateness of statistical tests used?**

Yes: I was able to assess this.

**Were you able to directly test the methods?**

Yes

**Comments to author:**

In this manuscript, Townes and Irizarry propose a new method for transforming read counts into UMI equivalents. This is done by learning transformation parameters from existing UMI datasets for which read count information is also available, and then creating a per-cell quantile mapping function to convert each cell's read counts to UMI counts. The proposed method is interesting and solves a somewhat important problem, given the large amounts of legacy read count data from published papers. However, I have a number of concerns about some of the theoretical aspects of the method as well as its applicability; these are described below in more detail.

1. The approach described by the authors involves forcing each cell's count distribution to a (cell-specific) target distribution. However, it is not clear to me that this addresses the actual problem of gene-specific amplification biases. One can expect, e.g., short transcripts to be more easily amplified, but this would not be resolved by a cell-centric quantile normalization.

Why not use a more obvious gene-centric approach where a quantile-quantile mapping from reads to UMIs for each gene is learnt from a training dataset and applied to the same gene in the test dataset? This would adjust for gene-specific effects while preserving the relative ranking of read counts across cells in the transformed QUMI data.

2. Confidence in the quantile mapping can be expected to drop as the read counts increase, simply because the parameter fit of the target distribution is dominated by the majority of low-abundance genes for each cell. How can a user be sure that any differential expression (or lack thereof) between cells is genuine and not an artifact of the normalization?

Library size normalization is simple enough that it is straightforward to diagnose why one cell has higher normalized expression values than another; this seems much more difficult for the authors' proposed strategy. One can imagine cases where a cell is genuinely highly upregulating a few genes, but that upregulation is dampened or lost because the corresponding large read counts are coerced into the target distribution.

3. I have a number of issues with the shape parameter:

- The authors claim that the probability of obtaining a count of zero from a Poisson-lognormal distribution depends only on the mean (Section 4.3.1). Well, that can't be right, as a few precursory simulations would reveal:

> mean(rpois(10000, 2^rnorm(10000, mean=2, sd=1))==0)

[1] 0.0593

> mean(rpois(10000, 2^rnorm(10000, mean=2, sd=4))==0)

[1] 0.2662

- The mapping procedure assumes that the shape value is constant for all cells. This does not seem reasonable to me - Figure 5 shows strong variation in the shape within and across datasets, and it seems that the shape may vary with the biological state of the cell (e.g., depending on how many genes are actively expressed).

4. Figure 8 is striking but seems to represent a relatively uncommon use case. In the absence of a clear biological signal, it is inevitable that the structure will be dominated by residual technical effects,

be they batch effects between tumors or variations in coverage across cells.

The authors should consider applying their method on more datasets, and in particular those that represent more typical applications of scRNA-seq technology, e.g., profiling populations of highly heterogeneous cell types. There should also be a clear demonstration of the improvement in the scientific/biological conclusions from each dataset - improved resolution of different tumors in the glioblastoma dataset is of little interest to me, this just represents a stronger batch effect.

**Reviewer 2**

**Were you able to assess all statistics in the manuscript, including the appropriateness of statistical tests used?**

Yes: I don't see major issues besides the comments provided in my review.

**Were you able to directly test the methods?**

Yes

**Comments to author:**

Unique molecular identifiers (UMIs) introduced in scRNAseq protocols can attenuate sources of technical noise, enabling the de-duplication of reads linked to the same molecule. Not all current scRNAseq protocols include UMIs. A prominent example is smart-seq2, which has been widely used to generate scRNAseq datasets. Townes and Irizarry introduce quasi-UMIs (QUMIs), a quantile normalization strategy for non-UMI scRNAseq data. Its aim is to transform the observed distribution of read-counts to match the distribution what would be observed if UMIs were used when generating the data. As a reference target, this transformation uses a Poisson-lognormal distribution whose parameter values match estimates obtained using external UMI scRNAseq datasets. The utility of the method is assessed by comparing QUMIs with UMIs in test datasets and illustrating the behaviour of QUMIs in the context of dimensionality reduction (PCA and GLM-PCA).

The idea of transforming non-UMI data into quasi-UMI data is interesting, as UMIs are known to reduce technical artefacts in scRNAseq datasets. As such, QUIMs might be of interest to those analysing or producing non-UMI data. However, some aspects of the analysis require further clarification (such as in terms of the choice of the shape parameter) and the assessment of how QUMIs interact with standard downstream analyses is limited.

Detailed comments are provided below.

Major comments

1. Abstract. Census counts are mentioned without introducing their definition or the relevant reference [1]. This concept is not necessarily known by potential readers and therefore I suggest the authors to edit the abstract accordingly.

2. Page 2, second paragraph. The authors indicate that the fraction of zeros per cell is a result of cell-to-cell differences in capture and RT efficiency, which has nothing to do with underlying biology. I believe this statement is not always accurate as there are situations in which the fraction of zeros could correlate with an underlying biological process. One example could be cell cycle, where one might expect the fraction of zeros to decrease as cells progress to later stages. I suggest to discuss this.

3. Figure 2 a-c. This figure illustrates a variable distribution of read counts (pre- and post- normalisation) when cells correspond to one cell type. As a comparison, it would be helpful to show similar plots for the same data, but based on UMI and QUMI counts. This will help to illustrate how UMIs and QUMIs can help to reduce this variability.

4. Section 2.3. Poisson-lognormal and Poisson-Lomax distributions are explored as a model for UMI counts. In the Methods, the authors discuss how the tail behaviour of this distribution compares with respect to the negative binomial distribution (which can be written as a Poisson-gamma mixture). As the negative binomial is a popular choice when modelling scRNAseq data, a useful comparison would be to also include a negative binomial fit to all panels in Figure 4.

5. Figure 4. The suitability of the Poisson-lognormal and Poisson-Lomax distribution as a model for UMI counts is supported using 3 (randomly?) selected cells from the Cao, Klein and Zeisel datasets. While this is a good illustration, it is does not show how this behaviour generalizes across cells. Perhaps the authors could report the distribution of a goodness-of-fit measure (BIC?) across cells for each model?

6. Choice of shape parameter. Figure 6 shows that the value of the shape parameter can affect the performance of QUMIs. As the inferred values for this parameter vary across training datasets (Figure 5), it would be good to understand if properties of the data can explain this variation. For example, are the inferred shape parameters correlated with the sparsity of the data? To assess this, it might be helpful to provide a scatterplot of estimated shape value versus the fraction of zeros/cell for each dataset. Moreover, as there is a large amount of publicly available UMI datasets, the training set could be expanded to illustrate how this variability generalises in a wider range of scenarios. The latter might help users to select an appropriate shape value for their data.

7. Figure 6. Please clarify whether boxplots represent the distribution of the estimated distances across cells or genes. Moreover, were the counts log-transformed prior to calculating the distance? Finally, while these distances are a good summary of the performance of the different normalisation approaches, it would best to understand how this behaviour varies across genes or cells? For example, the authors could include MA plots to explore the differences between UMI counts and QUMI/census counts depending on the underlying expression of a gene. Another aspect that could be assessed is whether QUMIs lead to similar variability estimates (such as CV2) than what would be obtained from UMIs. This could, for example, affect the highly variable gene selection step that is often applied prior to unsupervised clustering analyses.

8. Figure 7. The comparison between the top-left panel and the other panels is not direct as two aspects of the analysis change: the dimensionality reduction technique and the normalization strategy. A clearer comparison could be provided by applying PCA after the different normalizations as well as GLM-PCA for QUMIs and census. Moreover, as non-linear dimensionality reduction techniques (such as tSNE, UMAP) are often used in this context, potential users might also want to see the behaviour of QUMIs when using such methods. A similar comment applies to Figure 8. Is the reduction in the correlation with respect to the proportion of zeroes due to moving from PCA to GLM-PCA or due to the normalisation? Another helpful comparison would be to include standard global scaling normalisation techniques such as scran.

9. Reproducibility. It is great to see all software and analysis code available in GitHub. I suggest to also mention what specific software versions were used. For example, I was unable to run parts of the script as I had errors in the dependency to scran. The latter is most likely due to using a different software version.

10. Scalability. As scRNAseq datasets can be large, it would be helpful to also summarise the computational requirements for QUMIs and how does the method scale in terms of genes and or cells? How does that compare with respect to census counts?

Minor comments

1. Page 2, line 31. Replace "current protocols" by "some protocols".

2. Page 2, last paragraph. Replace "… log-normalised counts from … or SCnorm may …" by "… log-transformed counts after … or SCnorm normalisation may …".

3. Page 3, line 14. Add reference to [1] after "census counts method".

4. Figure 1, caption. "Each pane" -> "Each panel"

References

[1] Qiu et al (2017) Nature Methods. PMID: 28114287.

**Authors Response**

**Point-by-point responses to the reviewers’ comments:**

Reviewer #1:

1. The approach described by the authors involves forcing each cell's count distribution to a (cell-specific) target distribution. However, it is not clear to me that this addresses the actual problem of gene-specific amplification biases. One can expect, e.g., short transcripts to be more easily amplified, but this would not be resolved by a cell-centric quantile normalization.

Why not use a more obvious gene-centric approach where a quantile-quantile mapping from reads to UMIs for each gene is learnt from a training dataset and applied to the same gene in the test dataset? This would adjust for gene-specific effects while preserving the relative ranking of read counts across cells in the transformed QUMI data.

*We agree that gene-specific amplification bias is an important concern for full-length protocols. For this reason, we advocate for using TPM as input to our normalization instead of raw counts in section 2.6 “Quasi-UMIs enable dimension reduction of read counts” and the Conclusion section. We now also include the following sentence near the end of the introduction to further emphasize this important point: “When read counts are affected by gene-length bias, TPMs can be used as input to QUMI normalization.”*

*Could the reviewer please provide more detail about how to perform a gene-centric quantile mapping?*

*We attempted to do this but were not able to overcome the following obstacles (illustrated in the below figure)*

*1.	Unlike the distribution of expression values within a cell across genes (where we observed a canonical monotonically decreasing distribution reminiscent of a power law), when we examined the distribution of expression values for individual genes across cells we did not observe any common pattern that would allow us to specify a parametric family to use as a target distribution.*

*2.	We attempted to define a separate target distribution for each gene, but this also failed because even for the same gene the distribution was not consistent across datasets, even when the datasets (Zeisel and Klein) were from the same species.*

2. Confidence in the quantile mapping can be expected to drop as the read counts increase, simply because the parameter fit of the target distribution is dominated by the majority of low-abundance genes for each cell. How can a user be sure that any differential expression (or lack thereof) between cells is genuine and not an artifact of the normalization?

*Library size normalization is simple enough that it is straightforward to diagnose why one cell has higher normalized expression values than another; this seems much more difficult for the authors' proposed strategy. One can imagine cases where a cell is genuinely highly upregulating a few genes, but that upregulation is dampened or lost because the corresponding large read counts are coerced into the target distribution.*

*We now include a differential expression analysis on an additional dataset (Vieira Braga et al 2019) at the end of results section 2.5 “Quasi-UMIs approximate UMIs more closely than census counts” showing that p-values from QUMI counts are closer to the p-values computed from UMI counts than competing methods.*

3. I have a number of issues with the shape parameter:

- The authors claim that the probability of obtaining a count of zero from a Poisson-lognormal distribution depends only on the mean (Section 4.3.1). Well, that can't be right, as a few precursory simulations would reveal:

mean(rpois(10000, 2^rnorm(10000, mean=2, sd=1))==0)

[1] 0.0593

mean(rpois(10000, 2^rnorm(10000, mean=2, sd=4))==0)

[1] 0.2662

*We have now updated this section to emphasize that this is only true when the shape parameter sigma is fixed.*

- The mapping procedure assumes that the shape value is constant for all cells. This does not seem reasonable to me - Figure 5 shows strong variation in the shape within and across datasets, and it seems that the shape may vary with the biological state of the cell (e.g., depending on how many genes are actively expressed).

*We have now included four additional training datasets, and we do continue to see variation in the shape parameter estimates across different biological conditions (updated figure 5 in manuscript). We have now modified the results to reflect two possible approaches to QUMI normalization: (1) using a default shape parameter of 2.0 as before, and (2) selecting a more accurate shape parameter estimate by fitting Poisson-lognormal distributions to matched training data from the same tissue type as the test data (Table S1). We found that both approaches produce QUMI counts that are closer to UMI counts than census or read counts (updated Figure 6).*

*To further investigate the sensitivity of QUMI normalization to misspecifications of the shape parameter, we repeated the test data analyses with shape values of 1.0 and 3.0 (extremes of the MLEs from training data). While these misspecified values did generally degrade the accuracy, even in the worst case QUMI counts still outperformed census counts and read counts in terms of approximating the UMI counts.*

*Following the reviewer’s suggestion to compare shape parameters to cell-specific statistics, we plotted shape parameter estimates against the number of nonzero genes for each cell in all training datasets (see below). The correlations were strong in some datasets but weak in others, and the direction of the relationship was not consistent across datasets (positive in 3 datasets, negative in the four others). Based on this, we do not feel comfortable drawing any conclusions about the relationship between the shape parameter and biological state of the cell.*

4. Figure 8 is striking but seems to represent a relatively uncommon use case. In the absence of a clear biological signal, it is inevitable that the structure will be dominated by residual technical effects, be they batch effects between tumors or variations in coverage across cells. The authors should consider applying their method on more datasets, and in particular those that represent more typical applications of scRNA-seq technology, e.g., profiling populations of highly heterogeneous cell types. There should also be a clear demonstration of the improvement in the scientific/biological conclusions from each dataset - improved resolution of different tumors in the glioblastoma dataset is of little interest to me, this just represents a stronger batch effect.

*We now include an additional analysis at the end of the Results section of the pancreatic endocrine cells from Segerstolpe et al 2016 (a Smart-seq2 dataset). The original authors were unable to cluster 41 of the cells. Using QUMI counts, we were able to reclassify 20 of these unambiguously into known types (alpha, beta, delta, etc).*

Reviewer #2

1.	Abstract. Census counts are mentioned without introducing their definition or the relevant reference [1]. This concept is not necessarily known by potential readers and therefore I suggest the authors to edit the abstract accordingly.

*The abstract has now been edited.*

2.	Page 2, second paragraph. The authors indicate that the fraction of zeros per cell is a result of cell-to-cell differences in capture and RT efficiency, which has nothing to do with underlying biology. I believe this statement is not always accurate as there are situations in which the fraction of zeros could correlate with an underlying biological process. One example could be cell cycle, where one might expect the fraction of zeros to decrease as cells progress to later stages. I suggest to discuss this.

*We now changed this sentence to read “While the zero fraction could be driven by biological processes such as the cell cycle, this is completely confounded by cell-to-cell differences in capture and RT efficiency, which have nothing to do with underlying biology.”*

3.	Figure 2 a-c. This figure illustrates a variable distribution of read counts (pre- and post- normalisation) when cells correspond to one cell type. As a comparison, it would be helpful to show similar plots for the same data, but based on UMI and QUMI counts. This will help to illustrate how UMIs and QUMIs can help to reduce this variability.

*We now include UMI counts, QUMI counts, and scran normalizations in Figure 2.*

4.	Section 2.3. Poisson-lognormal and Poisson-Lomax distributions are explored as a model for UMI counts. In the Methods, the authors discuss how the tail behaviour of this distribution compares with respect to the negative binomial distribution (which can be written as a Poisson-gamma mixture). As the negative binomial is a popular choice when modelling scRNAseq data, a useful comparison would be to also include a negative binomial fit to all panels in Figure 4.

*We now include the negative binomial fits in Figure 4.*

5.	Figure 4. The suitability of the Poisson-lognormal and Poisson-Lomax distribution as a model for UMI counts is supported using 3 (randomly?) selected cells from the Cao, Klein and Zeisel datasets. While this is a good illustration, it is does not show how this behaviour generalizes across cells. Perhaps the authors could report the distribution of a goodness-of-fit measure (BIC?) across cells for each model?

*We now include BIC plots for all training data as supplemental figure S1. Unfortunately BIC could not clearly distinguish between the candidate models due to the preponderance of zeros and ones in the data. So, we developed an alternative goodness-of-fit assessment based on a predictive check that focuses on the tail of the distribution. As shown in supplemental figure S2, Poisson-lognormal has the best calibration to the data based on this criterion.*

6.	Choice of shape parameter. Figure 6 shows that the value of the shape parameter can affect the performance of QUMIs. As the inferred values for this parameter vary across training datasets (Figure 5), it would be good to understand if properties of the data can explain this variation. For example, are the inferred shape parameters correlated with the sparsity of the data? To assess this, it might be helpful to provide a scatterplot of estimated shape value versus the fraction of zeros/cell for each dataset.

*Please see the above scatter plot in our above response to a similar comment from the other reviewer. The relationship between detected genes and the shape parameter was unclear and inconsistent across datasets.*

*Moreover, as there is a large amount of publicly available UMI datasets, the training set could be expanded to illustrate how this variability generalises in a wider range of scenarios. The latter might help users to select an appropriate shape value for their data.*

*We now detail both a default and a custom approach to selecting the shape parameter. The latter suggests fitting MLEs to training data matched to the data of interest by having the same tissue type. We also performed a sensitivity analysis by using extreme values of the shape parameter and found that in all cases the QUMI counts were still more accurate than census or read counts.*

7.	Figure 6. Please clarify whether boxplots represent the distribution of the estimated distances across cells or genes. Moreover, were the counts log-transformed prior to calculating the distance?

*For each cell and each normalization we compute a single number metric which is the distance between the log of the normalized count vector and the log of UMI count vector (omitting zeros, which always match exactly for all normalizations). Each boxplot is then showing the distribution of these distance scores across all cells in each dataset.*

Finally, while these distances are a good summary of the performance of the different normalisation approaches, it would best to understand how this behaviour varies across genes or cells? For example, the authors could include MA plots to explore the differences between UMI counts and QUMI/census counts depending on the underlying expression of a gene. Another aspect that could be assessed is whether QUMIs lead to similar variability estimates (such as CV2) than what would be obtained from UMIs. This could, for example, affect the highly variable gene selection step that is often applied prior to unsupervised clustering analyses.

*We now include supplemental MA plots comparing all normalizations to UMI ground truth on two datasets. The first plot compares each gene’s average expression across cells and the second compares the coefficients of variation. These two criteria are both commonly used for feature selection (highly expressed and highly variable genes). The QUMI counts were most consistent with ground truth in all cases.*

8.	Figure 7. The comparison between the top-left panel and the other panels is not direct as two aspects of the analysis change: the dimensionality reduction technique and the normalization strategy. A clearer comparison could be provided by applying PCA after the different normalizations as well as GLM-PCA for QUMIs and census.

Moreover, as non-linear dimensionality reduction techniques (such as tSNE, UMAP) are often used in this context, potential users might also want to see the behaviour of QUMIs when using such methods. A similar comment applies to Figure 8. Is the reduction in the correlation with respect to the proportion of zeroes due to moving from PCA to GLM-PCA or due to the normalisation? Another helpful comparison would be to include standard global scaling normalisation techniques such as scran.

*We now include scran in both plots, as well as PCA applied to normalized counts for census and QUMI counts. UMAP plots are now included as a supplemental figure.*

9.	Reproducibility. It is great to see all software and analysis code available in GitHub. I suggest to also mention what specific software versions were used. For example, I was unable to run parts of the script as I had errors in the dependency to scran. The latter is most likely due to using a different software version.

*We now list the scran and monocle version numbers at the end of the first Methods section.*

10.	Scalability. As scRNAseq datasets can be large, it would be helpful to also summarise the computational requirements for QUMIs and how does the method scale in terms of genes and or cells? How does that compare with respect to census counts?

*We now discuss computational scalability at the end of Results section “Quantile normalization of read counts to quasi-UMIs”. Quasi-UMI normalization is somewhat faster than census, but they are on the same order of magnitude. Quasi-UMI normalization can be parallelized across cells to enable scaling to massive data, and we provide example code in the github repository.*

**Second round of review**

**Reviewer 1**

The paper is fine but the software needs much work. As of time of writing, the vignette is particularly uninformative, and I don't see any tests.

**Reviewer 2**

Apologies for the late review, but this has been a very busy period.

The authors have addressed most of the queries that I raised during the first submission of

this manuscript. However, I do have further comments regarding one of the new figures that

was provided as part of this revision. As part of my previous review, I suggested the following:

For example, the authors could include MA plots to explore the differences between UMI

counts and QUMI/census counts depending on the underlying expression of a gene. Another

aspect that could be assessed is whether QUMIs lead to similar variability estimates (such as

CV2) than what would be obtained from UMIs. This could, for example, affect the highly

variable gene selection step that is often applied prior to unsupervised clustering analyses.

*Response. We now include supplemental MA plots comparing all normalizations to UMI*

*ground truth on two datasets. The first plot compares each gene’s average expression*

*across cells and the second compares the coefficients of variation. These two criteria are*

*both commonly used for feature selection (highly expressed and highly variable genes). The*

*QUMI counts were most consistent with ground truth in all cases.*

Further comments. Many thanks for including these MA plots as I believe they enable readers

to better understand the performance of QUMIs and how it relates to gene-specific inference.

However, I believe that some aspects of this analysis still require further clarification.

1. How many genes were included in this analysis?

2. For scran, do the authors use the “quickCluster” step that is recommended when

analysing heterogeneous cell populations?

3. Figures S4 and S5 contain several thousands of genes and therefore it is not obvious

to visualise where most genes are concentrated. For example, are the most extreme

differences in the Figure mostly observed in a handful of genes? To facilitate this, I

suggest the authors to colour code the MA plots based on the density of data points.

4. The right hand side of the MA plots in Figure S4 suggests that the differences between

gene-level estimates obtained by the different methods are driven by a “global offset”

in which mean expression estimates are proportional to the ground truth estimates

that are derived from UMI data (this effect may be more prominent once densities are

provided). This offset might partially explain the differences in distances reported in

Figures 6 and S3. If this is the case, one may argue that the raw, scran normalised and

census counts fail to provide absolute expression values, but that relative expression

patterns are largely preserved (of course, this depends on how the density of genes is

distributed within the MA plot).

5. The most prominent differences reported in Figure S4 relate to lowly expressed genes

for which expression is likely captured in a small number of cells. Most of these genes

are typically removed before performing scRNAseq analyses and therefore the poor

behaviour observed in the left tails of the plots may have less practical consequences.

How do the MA plots look if a gene filtering step were to be applied to remove lowly

expressed genes (e.g. captured in less than 10% of the cells). Alternatively, the authors

could colour code the MA plots based on the percentage or number of cells for which

non-zero expression was capture for each gene. A similar analysis could be carried out

to better understand the results displayed in Figure S5.

6. Finally, a similar comment applies to the results of the DE analysis for the Vieira Braga

dataset (the authors include genes with non-zero counts in at least one cell, which is

a very liberal threshold). Are the differences in differences in results driven by lowly

expressed genes that were captured in a handful of cells?

**Authors Response**

**Point-by-point responses to the reviewers’ comments:**

Response to Reviewer 1

Reviewer #1: The paper is fine but the software needs much work. As of time of writing, the vignette is particularly uninformative, and I don't see any tests.

Response to Reviewer 2

1. How many genes were included in this analysis?

*18,378 genes for Tung and 14,175 genes for Zheng. The figure captions have now been updated with this information.*

2. For scran, do the authors use the “quickCluster” step that is recommended when

analysing heterogeneous cell populations?

*For the Patel dataset, yes we had already done this in the previous draft. For all other datasets (including the Zheng and Tung ones in the MA plots), we have now recomputed the scran normalization by using quickCluster first and updated all results and plots involving scran counts. As you can see in the manuscript, the plots are almost indistinguishable from before (where quickCluster was not used). This is because both Zheng and Tung data are relatively homogeneous so that the clustering doesn’t make a big difference in the size factor estimates of scran.*

3. Figures S4 and S5 contain several thousands of genes and therefore it is not obvious

to visualise where most genes are concentrated. For example, are the most extreme differences in the Figure mostly observed in a handful of genes? To facilitate this, I suggest the authors to colour code the MA plots based on the density of data points.

*We have now updated Figures S4 and S5 to show the density of genes in each hexagonal bin.*

4. The right hand side of the MA plots in Figure S4 suggests that the differences between gene-level estimates obtained by the different methods are driven by a “global offset” in which mean expression estimates are proportional to the ground truth estimates that are derived from UMI data (this effect may be more prominent once densities are provided). This offset might partially explain the differences in distances reported in Figures 6 and S3. If this is the case, one may argue that the raw, scran normalised and census counts fail to provide absolute expression values, but that relative expression patterns are largely preserved (of course, this depends on how the density of genes is distributed within the MA plot).

*We recomputed all M-A statistics using relative abundances from each normalization. As shown in the below graphs, QUMI counts are still closer to UMI counts than other normalizations even when compared on a relative rather than absolute scale. We would be happy to include these graphs in the supplemental figures if the reviewer recommends it. Note that both scran and census counts are basically indistinguishable from read counts in the relative abundance plots, since they are linear transformations of the read counts, whereas QUMI normalization is a nonlinear transformation.*

5. The most prominent differences reported in Figure S4 relate to lowly expressed genes for which expression is likely captured in a small number of cells. Most of these genes are typically removed before performing scRNAseq analyses and therefore the poor behaviour observed in the left tails of the plots may have less practical consequences. How do the MA plots look if a gene filtering step were to be applied to remove lowly expressed genes (e.g. captured in less than 10% of the cells). Alternatively, the authors could colour code the MA plots based on the percentage or number of cells for which non-zero expression was capture for each gene. A similar analysis could be carried out to better understand the results displayed in Figure S5.

*Please see below for the equivalents of figures S4 and S5 with genes detected in <10% of cells excluded. Note that in the Zheng dataset this threshold excludes 94% of the genes (ie, only about 900 out of 14,000 genes satisfied the filtering). For comparison, the Tung dataset had 12,000 out of 18,000 genes satisfy the filtering).*

*And here are the equivalents of the relative abundance MA plots with the same gene filtering applied.*

6. Finally, a similar comment applies to the results of the DE analysis for the Vieira Braga dataset (the authors include genes with non-zero counts in at least one cell, which is a very liberal threshold). Are the differences in results driven by lowly expressed genes that were captured in a handful of cells?

*Please see below where we have reproduced Fig S6 but this time excluding genes that were detected in <10% of cells. The ranking of methods is basically unchanged by the filtering, even though only about 1,000 out of the 12,000 genes met the threshold.*
